# Pattern of hospital antibiotic use in term neonates using WHO Access, Watch and Reserve Classification (AWaRe)

**DOI:** 10.12669/pjms.38.8.6098

**Published:** 2022

**Authors:** Muhammad Khalid, Javaria Rasheed, Iram Nawaz

**Affiliations:** 1 Dr. Muhammad Khalid, FCPS, Department of Pediatric Medicine, Nishtar Medical University Hospital Multan, Pakistan; 2 Dr. Javaria Rasheed, FCPS, Department of Pediatric Medicine, Nishtar Medical University Hospital Multan, Pakistan; 3 Dr. Iram Nawaz, FCPS, Department of Pediatric Medicine, Nishtar Medical University Hospital Multan, Pakistan

**Keywords:** Neonates, Sepsis, Antimicrobial stewardship, Pakistan, Antibiotics

## Abstract

**Background and Objective:**

Pakistan has high neonatal mortality rate and antibiotic utilization data in hospitalized term neonates from Pakistan is lacking. The study aimed to determine the pattern of hospital antibiotic use in term neonates using World Health Organization (WHO) Access, Watch, and Reserve classification (AWaRe).

**Methods:**

This cross-sectional chart review study was conducted at the neonatal unit of Pediatric Medicine Department of University Hospital over a period of one year from 1^st^ January 2020 to 31^st^ December 2020. Hospitalized full term newborns up to 28-days of life, of either gender were consecutively included in the study. Data on demographic characteristics, admission diagnoses and antibiotic prescribed (class, agent and duration) were extracted from clinical charts. Descriptive statistics in the form of mean ± SD and frequency and percentages were calculated.

**Results:**

A total of 2276 term neonates consisting of 69% (n=1570) males were included in the study. Antibiotic prescription rate was 92.8%. Most common reason for admission was birth asphyxia (36.1%) followed by sepsis (33%). Most commonly prescribed antibiotic Ampicillin (84%) belonged to Access group of WHO – AWaRe classification. More than 97% of prescriptions were two or more antimicrobial combinations belonging to Watch group. Ampicillin and Cefoperazone was the most commonly prescribed (42.8%) two drug combination. Meropenem and vancomycin was prescribed to 20% of the neonates – mostly for sepsis and pneumonia. Mortality rate was 7.7% in current study.

**Conclusion:**

High antibiotic prescription rate particularly from Watch-group is demonstrated in this study. There is high need of antimicrobial stewardship program in neonatal units.

## INTRODUCTION

Neonates and infants comprise majority of the six million annual deaths under-5 years of age around the world.^1^ The neonatal period is the most vulnerable time to infections due to immature immune system.^2^ In addition, exposure to invasive procedures,^3^ antibiotics use and cross-infections due to substandard nursing practices lead to alteration in normal microbial flora.^4^ Pakistan has the third highest neonatal mortality rate in the world.^5^ Neonatal infections is among the major causes of morbidity and mortality in neonates worldwide.^6^

The emergence of antibiotic resistance is one of the greatest challenges to the effective management of bacterial infections globally. Antimicrobial resistance has led to the longer durations of illness, increased treatment costs and high mortality rates.^7^ In low- and middle-income countries, this challenge is even greater, due to the higher burden of infectious diseases and the limited availability and affordability of antibiotics.^8^ The World Health Organization (WHO) recommends the development of context-specific, local essential medicine lists based on the local public health efficacy, safety and cost-effectiveness data of prescribed medicines.^9^

In 2017, the WHO Expert Committee on Selection and Use of Essential Medicines developed an instrument – The AWaRe classification of antibiotics, to be used at local, regional and global level for the monitoring of antibiotic utilization, setting the goals and assessing the impact of stewardship programs. Currently 258 antibiotics are included in this classification. The WHO has currently set a country level target of 60% antibiotic consumption from the Access class. Traffic light color codes have been suggested to indicate these categories – Access (green), Watch (amber), and Reserve (red).^10^

The present study aimed to document the antibiotic utilization in term neonates using WHO AWaRe classification in Pakistan to guide prioritization of future research with regards to rational antibiotic use in neonates.

## METHODS

This descriptive cross-sectional chart review study was conducted from 1^st^ January 2020 – 31^st^ December 2020 at Nishtar University Hospital Multan in the Neonatal unit of Pediatric Medicine Department. All consecutive charts of full term neonates (gestational age ≥ 37-weeks) between 1 – 28 days of life, who received at least one antibiotic during their hospital stay were included. Neonates admitted for post-operative care, syndromic features (Down’s, Edward syndrome) and major congenital malformations (i.e. congenital heart disease, trachea-esophageal fistula, diaphragmatic hernia and neural tube defects) were excluded from the study which was approved from institutional Ethics and Research Committee (ERC#1162, date 22-01-2022).

We created Excel 2016 spreadsheet as our master database. We extracted the initial data on total admissions, age (days), gender and diagnosis of the neonates during study period from admission / discharge register. We obtained the clinical charts of included neonates from the Medical Records and Statistical Department of the hospital for information on antibiotics used i.e. generic name, class, numbers, and duration of each antibiotic. All the data entry was supervised and double checked to minimize the bias. We used Statistical Package for Social Sciences (SPSS v. 23) for data analysis after importing the data from Excel spread sheet. The descriptive statistics were run in the form of mean ± standard deviation for numerical data and frequency and percentages for categorical data.

## RESULTS

A total of 2499 term neonates were admitted during study period. Of these neonates, 179 did not get any antibiotic and were excluded. Another 44 neonates with major congenital anomalies (n=25), syndromic features (n=7) and postoperative cases (n=12) were also excluded from the study. The final study group consisted of 2276 term neonates. Majority of the neonates were admitted within 24-hours of life (n=1058, 46.5%), 69% (n=1570) were males and 40.6 % (n= 923) were low birth weight. Most of the neonates were out born (87.9%, n=2000) and 78% (n=1776) of the deliveries occurred at healthcare facilities. Most common mode of delivery was normal vaginal delivery (58.6%, n=1334) and 54.1% (n=1231) were discharged from the hospital. Table-I.

**Table-I:** Characteristics of neonates admitted at Pediatric Medicine Department (N=2276).

Characteristics	Frequency	Percentages
***Age Groups***		
< 24-hours	1058	46.5
1-3 days	560	24.6
4-7 days	238	10.5
> 7-days	420	18.5
Birth weight		
Low birth weight (< 2500 grams)	923	40.6
Normal birth weight (≥ 2500 grams)	1353	59.4
***Gender***		
Males	1570	69
Females	706	31
***Inborn / Out born***		
Out born	2000	87.9
In born	276	12.1
***Place of delivery***		
Institutional deliveries	1776	78
Home delivery	500	22
***Mode of delivery***		
Normal Vaginal deliveries	1334	58.6
Cesarean section	942	41.4
***Outcome***		
Discharged	1231	54.1
Expired	176	7.7
Left against medical advice (LAMA)	869	38.2

Antibiotic prescription rate in term neonates over one year was 92.8%. Mean number of antibiotics prescribed / patient were 2.1 ± 0.5 and mean duration of antimicrobial therapy was 6.7± 5.1 days. Ampicillin was the most common antibiotic prescribed (n=1915, 84.1%) followed by Cefoperazone (n=986, 43.3%), Cefotaxime (n=922, 40.5%) and Meropenem (n=474, 20.8%). Antimicrobial days / 100 admission days were highest for Ampicillin (71) followed by Cefotaxime (37) and Cefoperazone (36) Table-II.

**Table-II:** Most common antibiotics prescribed to term neonates in neonatal unit according to WHO AWaRe classification (N=2276).

Antibiotics	Frequency	Percentage (%)	Antimicrobial days /100 admissions	AWaRe Class
Ampicillin	1915	84.1	71	Access
Cefoperazone	986	43.3	36	Watch
Cefotaxime	922	40.5	37	Watch
Meropenem	474	20.8	26	Watch
Vancomycin	455	20	25	Watch
Amikacin	61	2.7	3	Access

Only 2.4% of the neonates were treated with single antimicrobial agent. Most common single agent was Ampicillin (65.5%) followed by Cefotaxime (18.2%). More than three quarter of the neonates were prescribed two antibiotics. Most common of the two antibiotic combination was Ampicillin + Cefoperazone (42.8%) followed by Ampicillin + Cefotaxime in 35.9% of the neonates. Most common of the three drug combination was Ampicillin + Cefotaxime + Vancomycin in 18% followed by Ampicillin + Cefotaxime + Meropenem in 11.5%. Of all the four antibiotic combinations Ampicillin + Cefotaxime + Meropenem + Vancomycin was most common (36.0%). Table-III.

**Table-III:** Number of Antibiotic prescribed to term neonates (N=2276).

Antibiotic numbers	Antibiotics	Frequency (%)
Single antibiotic (n=55, 2.4%)	Ampicillin	36 (65.5)
	Cefotaxime	10 (18.2)
	Meropenem	08 (14.5)
Two antibiotics (n=1974, 86.7%)	Ampicillin + Cefoperazone	845 (42.8)
	Ampicillin + Cefotaxime	709 (35.9)
	Meropenem + Vancomycin	209 (10.6)
	Ampicillin + Meropenem	90 (4.6)
Three antibiotics (n=122, 5.4%)	Ampicillin + Cefotaxime + Vancomycin	22 (18)
	Ampicillin + Cefotaxime + Meropenem	14 (11.5)
	Ampicillin + Cefoperazone +Meropenem	13 (10.6)
	Ampicillin + Cefotaxime + Amikacin	10 (8.2)
Four antibiotics (n=125, 5.5%)	Ampicillin + Cefotaxime +	
	Meropenem + Vancomycin	45 (36.0)
	Ampicillin + Cefoperazone +	34 (27.2)
	Meropenem + Vancomycin	

Most common reason for admission was birth asphyxia (n=821, 36.1%) followed by sepsis (n=752, 33%) and neonatal jaundice (n=298, 13.1%). All the antibiotic combinations used to treat most common diagnoses comprised of Watch-category. Ampicillin + Cefoperazone was the most common antibiotic combination used in TTN (55.8%), JNN (47.3%), birth asphyxia (47.3%) and pneumonia (25%). This was second most common in meconium aspiration (38.6%) and third most common in sepsis (19.1%). Most common combination prescribed to neonates with sepsis and meconium aspiration was Ampicillin + Cefotaxime (23.9% and 41.2% respectively). Meropenem + Vancomycin was the second most commonly prescribed combination for sepsis (22.1%) and third most common for pneumonia (17.2%) Table-IV.

**Table-IV:** Antimicrobial prescription for six most common diagnoses in term neonates (N=2276).

Antibiotic Diagnosis	TTN (n=147)	Sepsis (n=752)	JNN (n=298)	Birth Asphyxia (n=821)	Meconium Aspiration (n=194)	Pneumonia (n=64)
Ampicillin	05 (3.4)	04 (0.5)	04 (1.3)	20 (2.4)	03 (1.5)	-
Ampicillin+Cefoperazone	82(55.8)	143(19.1)	141(47.3)	388(47.3)	75 (38.6)	16 (25)
Ampicillin + Cefotaxime	52(35.4)	180(23.9)	106(35.6)	278(33.9)	80 (41.2)	13 (20.3)
Ampicillin + Meropenem	-	53 (7.1)	05 (1.7)	22 (2.7)	08 (4.1)	02 (3.1)
Cefoperazone+Vancomycin	03 (2.1)	23 (3.1)	06 (2.1)	07 (0.9)	-	03 (4.7)
Cefotaxime + Vancomycin	-	20 (2.6)	06 (2.1)	04 (0.5)	01 (0.5)	05 (7.8)
Meropenem + Vancomycin	02 (1.4)	166(22.1)	06 (2.1)	21 (2.6)	06 (3.1)	11 (17.2)
Ampicillin+ Cefotaxime + Meropenem + Vancomycin	-	26 (3.5)	02 (0.7)	11 (1.3)	03 (1.5)	05 (7.8)

TTN: Transient tachypnea of newborn, JNN: Jaundice neonatorum.

## DISCUSSION

Our study documents a high prescription rate of antibiotics in neonates particularly from the Watch category. Concordant to our study high antibiotic prescription has been reported from countries like Rwanda (88%),^11^ Nepal (96%)^12^ and Zimbabwe (98%).^13^ However, a relatively low prescription rate has also been reported from India (71% in teaching hospital)^14^ and Iran (72%).^15^ This variation can be attributed to the characteristics of neonates as we did not include preterm neonates and neonates admitted in intensive care units. Other reasons for high prescription rate are higher number of the neonates born outside the institution and coming after third day of life.

Mean antibiotic duration in our study was lower than the study from University teaching hospital of Rwanda – 8.2 ±7.7 days.^11^ However, a study from Switzerland reported much lower antibiotic duration (median 4 (2-5) days).^16^ Higher duration of antibiotics in our study is due to the empirical nature of prescription, increased severity of illness due to delayed presentation and lack of standard antibiotic treatment guidelines and antibiotic stewardship program at our institution.

Ampicillin was highest prescribed antibiotic in our study from Access class. However, contrary to standard first line therapy in the form of Ampicillin and gentamicin, only 2.4% of the neonates in our study were prescribed Ampicillin alone and none received gentamicin. In more than 90% of the cases Ampicillin was combined with third generation Cephalosporin. Polypharmacy rate in our study was comparable to study from Nepal^12^ but higher than study from Iran reporting 63%.^15^ This pattern is against the WHO guidelines for using 60% of antibiotics from Access class^10^ and indicates lack of standard treatment protocols for empirical therapy in suspected neonatal infections.

Birth asphyxia was the most common admission diagnosis. However, in a study from Nepal neonatal sepsis was common admission diagnosis - 47.3%.^12^ Most common antibiotics used in neonates with birth asphyxia were Ampicillin + Cefoperazone and Ampicillin + Cefotaxime. Antibiotics utilized in a multicenter study of neonates with hypoxic-ischemic encephalopathy were ampicillin (87.9%), gentamicin (65.7%), and cefotaxime (37.2%).^17^ The infection rate in neonates with asphyxia is 20-40 times higher because antenatal infections, chorioamnionitis and maternal fever are potential factors of low Apgar score and need for resuscitation in delivery room.^18^

Meropenem and Vancomycin were most commonly prescribed to in sepsis and pneumonia. In a study from Kenya, most common antibiotic used for neonatal sepsis was penicillin and gentamicin.^19^ A local study from Bahawal Victoria hospital reported that 82.6% of neonates with sepsis were initiated with cefotaxime + amikacin.^20^ Meropenem prescription in our study was two times higher compared to 9% in a study from China^21^ and many folds higher than a study from USA^22^ (0.7%). Vancomycin use in our study was comparable to study 22.7%^12^ from Nepal but higher than study from Iran – 12%.^15^ These variations could be due to difference in study population, common pathogens isolated from the neonatal units, their antibiotic sensitivity patterns and number of sick neonates referred from secondary care hospitals. Neonatal mortality in our study was comparable to study from India – 7%^14^, Nepal – 6.4%^23^ and Rwanda – 10%.^24^ However, it was lower than the mortality reported from Cameroon – 15.7%.^25^ It could be due to the fact that we did not include preterm in our study and more than two third were institutional deliveries in our study.

### Limitations of study:

Being a single center study is one of our limitations. However, a large sample size over a period of one year and being the University hospital catering for the larger population, our study is likely to be representative of antibiotic use in term neonates. Due to small number of in-born neonates we did not separately compare the antibiotic utilization with out-born neonates.

## CONCLUSION

High antibiotic prescription rate particularly from Watch class is demonstrated in this study. Polypharmacy is observed in 90% of the cases. The study indicates lack of standard protocols for empirical treatment of neonatal infections. There is an urgent need for justified antimicrobial utilization in neonatal units considering the antibiograms of these units. Working healthcare professionals need to be trained on judicious antimicrobial use through antimicrobial stewardship program.

### Authors Contribution:

**MK:** Conceived, designed the study, did statistical analysis, editing of manuscript and final approval, responsible and accountable for the accuracy or integrity of the work.

**JR:** Did data collection, result interpretation, manuscript writing and critical review.

**IN:** Did data collection, literature review and wrote manuscript first draft.
